# Childhood socioeconomic position and adult leisure-time physical activity: a systematic review protocol

**DOI:** 10.1186/2046-4053-3-141

**Published:** 2014-12-05

**Authors:** Ahmed Elhakeem, Rachel Cooper, David Bann, Rebecca Hardy

**Affiliations:** MRC Unit for Lifelong Health and Ageing at UCL, 33 Bedford Place, WC1B 5JU London, UK

**Keywords:** Socioeconomic inequalities, Occupation, Education, Socioeconomic position, Physical activity, Exercise, Life course

## Abstract

**Background:**

Participation in leisure-time physical activity benefits health and is thought to be more prevalent in higher socioeconomic groups. Evidence indicates that childhood socioeconomic circumstances may have long-term influences on adult health and behaviour; however, it is unclear if this extends to an influence on adult physical activity. The aim of this review is to examine whether a lower childhood socioeconomic position is associated with lower levels of leisure-time physical activity during adulthood.

**Methods/design:**

Keywords will be used to systematically search five online databases and additional studies will be located through a search of reference lists. At least two researchers working independently will screen search results assess the quality of included studies and extract all relevant data. Studies will be included if they are English language publications that test the association between at least one indicator of childhood socioeconomic position and a leisure-time physical activity outcome measured during adulthood. Any disagreements and discrepancies arising during the conduct of the study will be resolved through discussion.

**Discussion:**

This study will address the gap in evidence by systematically reviewing the published literature to establish whether childhood socioeconomic position is related to adult participation in leisure-time physical activity. The findings may be used to inform future research and policy.

**Systematic review registration:**

PROSPERO CRD42014007063.

**Electronic supplementary material:**

The online version of this article (doi:10.1186/2046-4053-3-141) contains supplementary material, which is available to authorized users.

## Background

Regular physical activity has positive effects on health and mental well-being and promotes independent living in later life, while inactivity on the other hand accrues public health costs that are comparable to smoking [1]. Evidence suggests that leisure-time physical activity (LTPA) is more strongly and consistently associated with health outcomes than other domains of activity [2]. LTPA also makes up the majority of time spent in moderate to vigorous intensity physical activity [3], may be easier to maintain than other domains [4], and is potentially more amenable to intervention across all life stages (e.g. occupational physical activity interventions have limited use in retired populations). However, a better understanding of the lifetime correlates and determinants of LTPA is required for the development of targeted evidence-based interventions.

Like many health outcomes and behaviours, LTPA is associated with contemporaneous socioeconomic circumstances; evidence from existing systematic reviews indicates that less socioeconomically advantaged adolescents [5] and adults [6] tend to participate in less LTPA compared with their more advantaged peers. However, inconsistencies in these associations have been described [5, 6], and the finding in one review [6] that some indicators of socioeconomic position (SEP), such as educational attainment, appeared to be more strongly related to physical activity than others was not replicated by a later review [7].

In addition to the well-documented associations between adult SEP and adult health and related behaviours, a substantial body of evidence suggests long-term influences of childhood socioeconomic circumstances on adult health and behavioural outcomes [8]. These associations are typically of substantial magnitude and are not fully explained by the continuity of socioeconomic circumstances from childhood into adulthood [8]. However, studies specifically linking childhood SEP with adult physical activity are inconsistent [9], and the existing literature has not yet been systematically reviewed.

The aim of this review is to assess whether an association exists between childhood SEP and adult LTPA by testing the hypothesis that a lower childhood SEP is associated with less frequent participation in LTPA during adulthood. As secondary objectives, the review will attempt to determine the strength of this relationship, examine the extent to which any association is explained by the continuity of SEP from childhood into adulthood and explore sources of between-study heterogeneity including the use of different indicators of childhood SEP.

## Methods/design

### Protocol registration

This study protocol is registered with the PROSPERO database (registration number: CRD42014007063).

### Eligibility criteria

Studies will be included if they:

- Test the association between at least one indicator of childhood SEP (i.e. that indicate SEP at any age up to age 18) and an adulthood LTPA outcome (measured from age 25).
- Are published in a peer-reviewed journal.
- Are reported in English.
- Include population-based samples.
- Use any observational study design, e.g. prospective cohort, retrospective cohort, case-control study.

Eligible indicators of childhood SEP are any recognised resource or prestige-based measure of position within a societal structure [10] referring to participants’ early life (e.g. parental occupation, parental education, family income, housing characteristics, and indices combining multiple indicators). Both recalled and prospectively ascertained measurements of childhood SEP will be eligible for inclusion. Own education will not be considered an eligible exposure despite its occasional use as an indicator of childhood SEP as it also captures the influence of adult resources [11].

All outcomes capturing physical activity performed during free time for personal interest will be considered including: sport, i.e. structured, organised and often competitive physical activity; exercise, i.e. planned, repetitive and purposeful physical activity; and total LTPA [12]. We selected a minimum 7-year period between ascertainment of childhood SEP and adult LTPA as we are specifically interested in exploring the long-term influences of childhood SEP. The minimum age of 25 at measurement of LTPA should in addition allow us to inspect whether any associations are explained by the continuity of SEP from childhood to adulthood in studies that additionally adjust for own adult SEP.

Reviews, unpublished literature, studies measuring strictly non-LTPA outcomes (e.g. occupational activity only) and those with unrepresentative samples (e.g. hospital inpatients, care home residents) will be excluded.

### Search strategy

**Figure 1:**
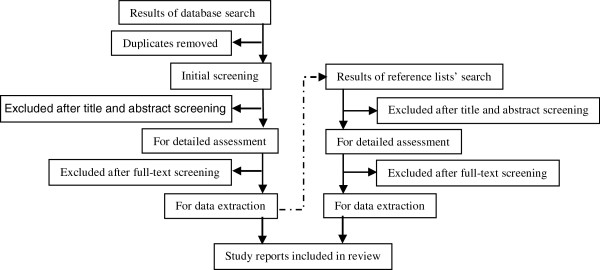
**PRISMA study flow chart.**

The following five online databases will be searched systematically using free-text synonym keywords to locate all eligible articles available up to the date of the final search: Embase (from 1974), MEDLINE (from 1946) and PsycINFO (from 1806) via the OvidSP interface and CINAHL (from 1937) and SPORTDiscus (from 1985) via the EBSCO interface (Table 1). Search terms will be tested in preliminary trials to improve the effectiveness of the final search. Proximity and Boolean logic operators and truncation commands will be used when executing the search and will be modified where necessary for each interface (Table 2). A search of the included papers’ reference lists will supplement the electronic database search (Figure 1).

**Table 1:** **EBSCO (CINAHL and SPORTDiscus) search strategy**

	Search terms
1.	(physical* activ*)
2.	(leisure N3 time)
3.	(sport*)
4.	(exercise)
5.	(walk*)
6.	(recreational)
7.	(father* N3 (occupation* or education*))
8.	(mother* N3 (occupation* or education*))
9.	(parent* N3 (occupation* or education*))
10.	(father* N3 (income or manual))
11.	(mother* N3 (income or manual))
12.	(parent* N3 (income or manual))
13.	(father* N3 (social class or social status))
14.	(mother* N3 (social class or social status))
15.	(parent* N3 (social class or social status))
16.	(child* N3 (social class or social status))
17.	(early-life N3 (social class or social status))
18.	(adolescen* N3 (social class or social status))
19.	(father* N3 (socioeconomic or socio-economic))
20.	(mother* N3 (socioeconomic or socio-economic))
21.	(parent* N3 (socioeconomic or socio-economic))
22.	(child* N3 (socioeconomic or socio-economic))
23.	(adolescen* N3 (socioeconomic or socio-economic))
24.	(early N3 (socioeconomic or socio-economic))
25.	(early-life N3 (socioeconomic or socio-economic))
26.	(child* N3 (deprivation or poverty))
27.	(early-life N3 (deprivation or poverty))
28.	(adolescen* N3 (deprivation or poverty))
29.	(child* N3 overcrowding)
30.	(adult*)
31.	(midlife or mid-life)
32.	(old*)
33.	(later-life)
34.	1 OR 2 OR 3 OR 4 OR 5 OR 6
35.	7 OR 8 OR 9 OR 10 OR 11 OR 12 OR 13 OR 14 OR 15 OR 16 OR 17 OR 18 OR 19 OR 20 OR 21 OR 22 OR 23 OR 24 OR 25 OR 26 OR 27 OR 28 OR 29
36.	30 OR 31 OR 32 OR 33
37.	34 AND 35 AND 36
38.	Remove duplicates from 37

'*' is a search command for capturing different word endings - see Table 2 for more details.

**Table 2:** **Tools and techniques to be used in the online database search**

Tool/technique	Description	Example
Synonyms keyword search	Search using all known synonyms including both British and US spellings.	Socioeconomic position synonyms: socioeconomic, socio-economic, occupation, education etc.
Truncation commands	'root word*': captures alternative word endings	Occupation*: searches for occupation, occupations, occupational etc.
Proximity operators	Identify words that are within a chosen distance of each other. Operators used will be Adj3 in OvidSP interface and N3 in EBSCO interface.	Occupation* adj3 class*: locates articles where occupation* and class* are within three words of each other.
Boolean logic operators	Two commands will be used	
	1. ‘OR’ to locate results with at least one of the search terms present.	1. (Occupation* OR education*): returns results with occupation* or education*.
	2. ‘AND’ will be used near end of search to combine results of different search concepts.	2. Concept 1 (e.g. childhood SEP) AND concept 2 (e.g. adult LTPA): returns results that include concepts 1 and 2.

### Study selection

Results of the database searches will be merged and abstracts screened by two researchers (from AE, RC and RH) working independently. Duplicate citations will be identified and removed using database filters prior to merging results and by using the ‘Duplicate Search’ command in Reference Manager 12 after merging. Full texts of potentially eligible papers will be double screened, and all exclusions including reasons for exclusion will be noted (see Additional file 1). Disagreements will be resolved through discussion between AE, RC and RH.

### Data extraction

The following information will be extracted from all included papers: citation details including title and year of publication, study details including design, country/region and sample size, exposure and outcome details including type of indicators used and how and when these were ascertained, participant details including age and sex, statistical methods used, e.g. logistic regression analysis, information on adjustment for potential confounding and mediating factors and lists of potentially eligible papers identified from reference lists. We will extract all statistics relating to the association of interest, i.e. difference in prevalence or odds ratios for a binary LTPA outcome, correlation coefficients, mean differences or regression coefficients for a continuous LTPA outcome and related measures of precision, i.e. 95% confidence intervals or standard errors. We will test the data extraction form (see Additional file 2) on a sample of included papers and modify it as required. All data will be double extracted (by AE, RC, DB and RH), and discrepancies will be resolved through discussion between all four extractors.

### Quality assessment

We will assess the quality of each included study concurrently with data extraction using a version of the Newcastle-Ottawa scale [13] which will be modified as recommended to ensure the capture of all relevant information [14]. Study quality will be judged based on the following criteria: representativeness, adjustment for covariates, length of follow-up and methodology used to measure childhood SEP and adult LTPA (see Additional file 3). Quality scores will be calculated as the average of two reviewers’ ratings with a potential range from 0 (lowest quality score) to 9 (highest quality score). Quality rating will not be used to exclude studies and will instead be integrated into the synthesis of the findings. In this way, quality assessment scores will help identify studies whose results may have been influenced by aspects of their methodology and/or design. The quality assessment forms will be piloted on a subsample of included papers and refined as necessary.

### Synthesis

Tables will be used to summarise the characteristics and results of included studies. If sufficient consistency in the method of reporting results is found between studies, e.g. if several studies report comparable odds ratios or differences in means, then random effects meta-analyses will be used to pool effect estimates allowing us to give a summary measure of the overall strength of association. Subgroup meta-analysis or meta-regression will be used to explore three *a priori* selected sources of between-study heterogeneity: a) type of childhood SEP indicator, b) region/country (to examine cultural and country income level influences) and c) age at LTPA assessment. We will investigate the extent to which any associations are explained by the continuity of SEP from childhood to adulthood by pooling adjusted estimates of studies that additionally adjust for adult SEP and will compare these to an unadjusted pooled estimate.

Where two or more papers report on the same study then only one of these would be included in the meta-analysis. Results will be presented graphically in forest plots, and the extent of heterogeneity will be assessed using Cochran’s Q statistic [15] and Higgins and Thompson’s *I*^2^ [16]. Sensitivity analyses will test whether any one study is explaining the observed heterogeneity by excluding each study in turn. Evidence of publication bias will be assessed via funnel plots.

### Reporting

The review and its findings will be reported in accordance with the Preferred Reporting Items for Systematic Reviews and Meta-Analysis (PRISMA) statement [17].

## Discussion

This study will systematically review the published literature to determine whether an association exists between childhood socioeconomic position and adult leisure-time physical activity. The relationship between different indicators of childhood SEP and adult LTPA and other sources of between-study heterogeneity will be investigated. We will consider the strengths and limitations of the identified evidence as well as those of our review, and we will discuss the findings in the context of other relevant reviews. In addition to addressing the gap in evidence surrounding the topic area, findings of this review could provide a framework for future research and may inform the design of physical activity behaviour change or social policy interventions.

## Authors’ information

AE is investigating how socioeconomic and biological early life factors might relate to adult physical activity as part of an MRC PhD studentship in life course epidemiology. RC, DB and RH supervise the research.

## Electronic supplementary material

Additional file 1: **Study selection form.** The data contains the checklist to be used for screening potentially eligible papers for inclusion in the review. (PDF 46 KB)

Additional file 2: **Data extraction form.** The data contains the form to be used by each assessor for extracting relevant data such as exposure/outcome details and results from the included studies. (PDF 115 KB)

Additional file 3: **Quality assessment form.** The data contains a checklist based on an amended version of the Newcastle-Ottawa scale which is to be used for grading the quality of included studies. (PDF 59 KB)

## Abbreviations

LTPA: Leisure-time physical activity
SEP: Socioeconomic position
PRISMA: Preferred reporting items for systematic reviews and meta-analysis.
