# Supplementary material for: Childhood socioeconomic position and adult leisure-time physical activity: a systematic review protocol
Source: Syst Rev. 2014 Dec 5;3:141. doi: 10.1186/2046-4053-3-141 (PMC4265411; doi:10.1186/2046-4053-3-141)
Supplement: Supplementary file 1 — Additional file 1: Study selection form. The data contains the checklist to be used for screening potentially eligible papers for inclusion in the review. (PDF 46 KB) [file 13643_2014_308_MOESM1_ESM.pdf]

A. Reference details	
A1. Reference Manager ID number (Ref ID)	
A2. 1st Author	
A3. Title of paper	
A4. Journal, volume, year of publication	
A8. Assessor's name, date of assessment	

B. Eligibility				
B1. Study included in systematic review?	Yes		No	

C. Reason(s) for exclusion (if excluded)				
C1. Outcome not in adults (≥ 25 yrs.)	Yes		No	
C2. Ineligible exposure	Yes		No	
C3. Ineligible outcome	Yes		No	
C4. Review article	Yes		No	
C5. Duplicate (Insert Ref ID of other study)	Yes<br>Ref ID:		No	
C6. Other				
