# Supplementary material for: Childhood socioeconomic position and adult leisure-time physical activity: a systematic review protocol
Source: Syst Rev. 2014 Dec 5;3:141. doi: 10.1186/2046-4053-3-141 (PMC4265411; doi:10.1186/2046-4053-3-141)
Supplement: Supplementary file 2 — Additional file 2: Data extraction form. The data contains the form to be used by each assessor for extracting relevant data such as exposure/outcome details and results from the included studies. (PDF 115 KB) [file 13643_2014_308_MOESM2_ESM.pdf]

A. Reference details								
A1. Ref ID, 1st author, title, publication year,								
A2. Assessor's name and date of assessment								
B. Study details								
B1. Name of study/cohort								
B2. Design	Prospective cohort		Retrospective cohort		Case-control		Other	
B2A. If other:								
B3. Country, setting								
C. Childhood socioeconomic position								
C1. Parental occupation	Yes						No	
C2. Parental education	Yes						No	
C3. Other measures (list):								
C4. How ascertained	Prospectively						Retrospectively	
C5. Age recorded								
C6. Age referred to								
D. Physical activity (PA) outcomes								
D1. Parameters measured	Frequency		Type		Duration		Intensity	
D2. Type of leisure-time PA (LTPA) measured	Sport/ exercise		Gardening/ DIY		Total LTPA		Other	
D2A. If other please describe								
D3. How ascertained	Self-reported						Objective methods	
D4. Age ascertained								
D5. Variable details	Binary		Ordinal		Continuous		Other	
D5A. If other:								
E. Available participant numbers								
E1. Baseline	Yes		No				If yes, number	
E2. Excluded	Yes		No				If yes, number	
E3. Refused	Yes		No				If yes, number	
E4. Lost to follow-up	Yes		No				If yes, number	
E5. Other losses	Yes		No				If yes, number	
E6. Included in analysis	Yes		No				If yes, number	
E7. All accounted for	Yes		No					
F. Analysis								
F1. How results analysed	Descriptive/ Trend		Logistic regression		Linear regression		Other	
F1A. If other:								
F2. Included in analysis	Men and women		Men only				Women only	
F3. Only significant results presented	Yes						No	
G. Summary of results								
G1. Prevalence/Mean difference	Yes						No	
G2. Odds/Risk ratios	Yes						No	
G3. Regression coefficients	Yes						No	
G4. Confidence intervals (CIs)/ P-value/standard errors (SE)	Yes						No	
G5. Other	Yes						No	
G5A. If other								
H. References for screening								
H1. Reference numbers								

I. Effect estimates

Association tested	Number analysed	Type of effect estimate and category comparison/value of unit change	Effect estimate	95% CI; SE; p-value	Confounders included in analysis
1.					
2.					
