# Supplementary material for: Childhood socioeconomic position and adult leisure-time physical activity: a systematic review protocol
Source: Syst Rev. 2014 Dec 5;3:141. doi: 10.1186/2046-4053-3-141 (PMC4265411; doi:10.1186/2046-4053-3-141)
Supplement: Supplementary file 3 — Additional file 3: Quality assessment form. The data contains a checklist based on an amended version of the Newcastle-Ottawa scale which is to be used for grading the quality of included studies. (PDF 59 KB) [file 13643_2014_308_MOESM3_ESM.pdf]

Note: A study can be awarded a maximum of two stars for each numbered item – (except number 5).

A. Selection

1) Representativeness of the exposed cohort
- a) Truly representative of the source population. ✸ ✸
- b) Somewhat representative of the source population. ✸
- c) Selected group of users e.g. nurses, volunteers.
- d) No description of the derivation of the cohort.

2) Ascertainment of childhood socioeconomic position (SEP)
- a) Prospectively from parents/participants when aged ≤18 years. ✸✸
- b) Retrospectively collected with attempts to reduce recall bias (e.g. life-grid and structured interview techniques). ✸
- c) Retrospectively collected without attempts to reduce recall bias.
- d) No description.

B. Comparability

3) Comparability of cohorts on the basis of the design/analysis
- a) Study controls for adult SEP. ✸
- b) Study controls for any additional relevant factors (e.g. age, sex). ✸
- c) Only unadjusted model presented.

C. Outcome

4) Assessment of physical activity
- a) Objective methods (heart-rate monitoring/accelerometer). ✸ ✸
- b) Self-reported using validated questionnaire/diary/interview. ✸
- c) Self-report.
- d) No description.

5) Adequacy of cohort follow-up
- a) Complete follow up - all subjects accounted for. ✸
- b) Subjects lost to follow up unlikely to introduce bias (≥75% follow-up or description provided of those lost). ✸
- c) <75% follow-up and no description of those lost.
- d) No statement.
